# Global Health Perspectives on Cigarette Butts and the Environment

**DOI:** 10.3390/ijerph16101858

**Published:** 2019-05-26

**Authors:** Paula Stigler Granados, Lawrence Fulton, Evangelina Nunez Patlan, Mischa Terzyk, Thomas E. Novotny

**Affiliations:** 1 School of Health Administration, Texas State University, San Marcos, TX 78666, USA; lf25@txstate.edu (L.F.); e_n90@txstate.edu (E.N.P.); 2 Framework Convention Alliance (FCA) for Tobacco Control, Ottawa, ON K1N 7B7, Canada; terzykm@fctc.org; 3 School of Public Health, San Diego State University, San Diego, CA 92106, USA; tenovotny@gmail.com

**Keywords:** tobacco product waste, framework convention, cigarette butts, tobacco control

## Abstract

Cigarette butts, which are also known as tobacco product waste (TPW), are the single most collected item in environmental trash cleanups worldwide. This study used an online survey tool (Qualtrics) to assess knowledge, attitudes, and perceptions regarding this issue among individuals representing the Framework Convention Alliance (FCA). The FCA has about 680 members on its listserv, including non-governmental tobacco control advocacy groups that support the implementation of the World Health Organization’s (WHO) Framework Convention on Tobacco Control (FCTC). Respondents (*n* = 65) represented countries from all six WHO regions. The majority (82%) had heard the term TPW, and they all considered TPW as an environmental harm at some level. Additionally, 29% of respondents failed to identify that “cigarette filters make smoking easier”. Most (73%) correctly identified TPW components; however, fewer (60%) correctly identified the composition of cigarette butts. The majority (57%) were unfamiliar with Extended Producer Responsibility (EPR) and Product Stewardship (PS) as possible environmental intervention strategies. Respondents expressing opinions concurred that adding a litter fee to fund TPW programs will aid in reducing tobacco use and reduce the environmental impacts of TPW (100%); that prevention, reduction, and mitigation of TPW could be an important part of international tobacco control programs (98%); and, that banning smoking in outdoor venues could reduce TPW (95%). Only 16% reported effective prevention or clean-up efforts in their countries. Weighted rankings revealed that respondents’ saw the national government, the tobacco industry, and state governments as the most important in addressing TPW. The results of this research will inform continuing international discussions by the FCTC Conference of the Parties (COP) regarding environmental policies that may be addressed within FCTC obligations.

## 1. Introduction

Tobacco use is not only a public health threat, it is also a major environmental issue. While research suggests that smoking accounted for 11.5% of global deaths in 2015 [1], our knowledge of the environmental externalities of tobacco production and consumption is less understood. Several organizations, including the World Health Organization (WHO), have acknowledged the environmental harm caused by tobacco use and its production [2,3,4]. Deforestation and the loss of biodiversity, exposure to hazardous chemicals used during cultivation and manufacturing, and the waste from cigarette butts are all direct environmental consequences of the lifecycle of tobacco [5,6]. Almost six-trillion cigarettes are produced globally each year, with approximately one-third to two-thirds of those cigarette butts being possibly deposited in the environment and ending up in parks, beaches, streets, and communities [2,7].

Cigarette butts containing toxic chemicals are the leading item collected during environmental cleanups around the globe [8,9,10]. There is an estimated 766,571 metric tons of cigarette butts that are annually deposited into the environment [2,7]. It is common for tobacco product waste (TPW) to be improperly discarded due to social norms that are associated with the smoking ritual along with an increase in indoor smoking bans that push smokers outside [11]. Cigarette filters are made of paper and cellulose acetate, which is a nearly non-biodegradable plastic that collects chemicals that are produced by smoking [12]. This plastic component of filtered cigarettes may not degrade in the environment for many years. Even after deterioration, TPW may persist as small particles of toxic-infused plastic waste, which can leach into soil and water supplies [13]. Slaughter et al. showed that one smoked cigarette butt soaked in a liter of water for 96 h reached the Lethal Concentration 50 (LD 50) for test fish that were exposed to the leachates [14]. TPW is harmful, and there is an abundance of it found in the environment across the planet [15].

Healthcare costs are only one of the many economic and social costs of tobacco [16]. The life cycle costs from tobacco production to disposal must be considered. Some of these costs are associated with growing, curing, manufacture, production, distribution, transportation, consumption, and post-consumption/disposal [3,17]. The post-consumption stage of tobacco involves multiple levels of responsibility [18].

The development of deposit/return/take-back programs, labeling cigarettes as hazardous waste, applying litter fees to tobacco sales, suing entities for clean-up and nuisance costs, fining individuals for littering, banning filtered cigarette sales, and educating consumers are amongst the recommended methods for preventing and mitigating TPW [19]. Anti-littering legislation is the most common strategy for TPW mitigation; however, these laws target the individual smoker and burden law enforcement officials. Further, enforcement and prevention are often minimal, so such legislation does not prevent TPW from entering the waste stream [12,20]. These types of regulatory efforts are demand-side, downstream approaches that hardly address the problem of global TPW [21]. Research suggests a need to emphasize environmental principles, such as Extended Producer Responsibility (EPR) and Product Stewardship (PS) [22]. EPR makes the product manufacturer responsible for the entire lifecycle waste stream of their product. PS calls for shared responsibility by all parties that are involved in the distribution and use of the product [23]. Just as other industries are responsible for their waste (e.g., paint, tires, electronics, etc.), the tobacco industry should be held accountable for TPW and policies created to assist with this process.

The Framework Convention on Tobacco Control (FCTC), which was founded in February 2005, consists of 181 Parties and it is the first health treaty enacted under the authority of the World Health Organization (WHO) [24,25]. Articles 9, 18, and 19 of the Convention address tobacco-related environmental issues and tobacco industry responsibility for harms [25]. The Framework Convention Alliance (FCA) is a collection of civil society groups that support the development, ratification, implementation, and monitoring of the FCTC [26]. The FCA plays a vital role in the implementation process for the FCTC.

EPR and PS principles may apply to TPW under Articles 9, 18, and 19. FCA members were previously surveyed in 2014 to assess knowledge, attitudes, and perceptions regarding TPW [8]. This study is the second such assessment of the same group in order to determine if there have been any changes in knowledge and perceptions and to provide evidence on how best to support next steps in future policy work on this issue. The findings from the previous study may be generally compared with those of this study. The WHO is considering additional research and policies regarding the lifecycle environmental impacts of tobacco use, tobacco agriculture, tobacco manufacturing, and TPW [25]. The Cigarette Butt Pollution Project (CBPP), which is a non-profit organization registered in California and a member of the FCA, conducted the study in collaboration with San Diego State University and Texas State University researchers.

## 2. Materials and Methods 

The study population was a convenience sample of FCA members that was obtained through the online survey tool, Qualtrics [27]. The email listserv of 683 current FCA members was provided to CBPP by the FCA Secretariat in Ottawa, ON. Data collection was completed from January 14 through February 28, 2019. The survey had three sections: (1) demographic information about participants and their role in their organization/country, (2) knowledge and beliefs regarding TPW, and (3) awareness and perceptions towards TPW and related environmental principles. Questions were previously developed based on published TPW studies, such as by Rath et al. and they were similar to those that were used in the previous study [8,11]. The online survey was administered according to FCA communication protocols. The study was approved by the Institutional Review Board (IRB) of San Diego State University (HS-2018-0204). No incentives were offered for participation, and an informed consent statement was provided upon the beginning the survey, which indicated the voluntary and confidential nature of the study. The information collected was confidential but not anonymous, as we were interested in the types of organizations and membership status of participants. No explanations were provided about environmental principles queried (e.g., EPR and PS) in order to ascertain basic knowledge about these principles among the respondents. The respondents to this survey provided individual-level responses rather than institutional positions. The initial survey was sent out on January 14 and it was made available in English, Spanish, and French, with five subsequent reminder emails beinf sent to the listserv requesting participation.

We used descriptive analysis to evaluate the overall knowledge, awareness, and perceptions of the participants regarding TPW. Missing responses or “I don’t know” were classified as incorrect. Inferential analysis evaluated knowledge versus demographics (gender, country region, age, years worked). 

One of the primary dependent variables of interest was the proportion of correct responses on the 17 knowledge questions (quantitative, ratio). Other dependent variables, those demonstrating high variability, were Likert-scaled (e.g., “Cigarette filters make cigarettes easier to smoke”) and were thus treated as ordinal for analysis. R statistical software [28] was used to analyze the data, along with several R packages.

## 3. Results

### 3.1. Demographics

**Figure 1:**
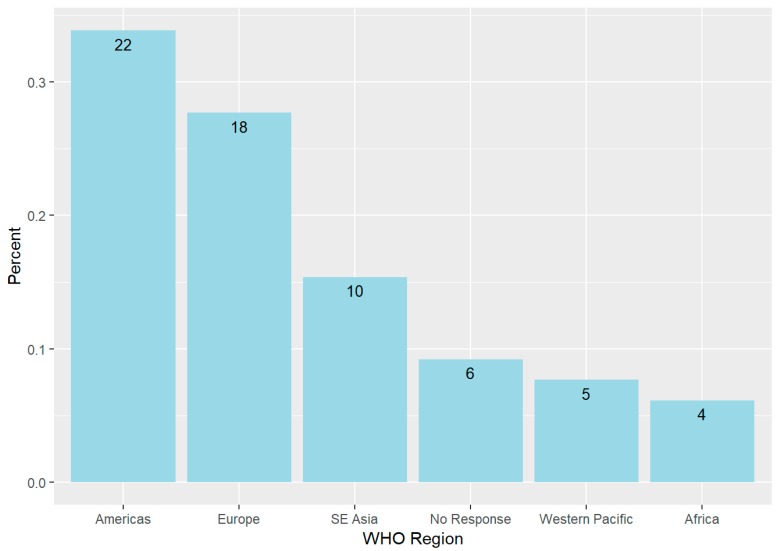
WHO Regions Represented by Framework Convention Alliance Survey Participants, 2019.

The response rate was 10% (*n* = 65). The respondents represented countries from five of six WHO regions: Americas (33%), Europe (27%), Southeast Asia (15%), Western Pacific (7%), Africa (6%), and 9% provided no response to their country of origin. The participants represented 37 countries. All of the participants were 31 and older, with 48% reporting that they were over the age of 50 years. The gender distribution was relatively balanced, with 31 males, 28 females, and six non-disclosures. Most participants (76%) had worked ≥10 years in tobacco control. Few (20%) were involved in any environmental groups. Most were from the Americas (22) and Europe (18) (Figure 1).

### 3.2. Knowledge and Beliefs

The mean percentage correct for all TPW knowledge questions was 84% (+/−11%). While all of the subjects correctly identified cigarette butts as TPW, only 60% correctly identified ashtrays and electronic waste as TPW (Table 1).

Regarding knowledge of the most common items that are picked up on beach and roadway cleanups, 82% identified the correct response (TPW) with the remaining 18% identifying plastics (e.g., bags, straws, bottles, cups); none identified fishing line, nets, bottles, or cans.

The majority correctly understood that cigarette filters are not generally considered to be biodegradable (88%), that they do not make cigarettes less harmful to smoke (98%), and that discarded cigarette butts are toxic waste products (92%). However, only 71% of the respondents knew that cigarette filters make it easier to smoke. The mean percentage correct on all of the questions was 87%.

The respondents were asked about the composition of cigarette filters. Options included plastic, plastic, and paper, as well as cotton, cork, and other. The respondents were able to write-in any material that they believed to be part of a cigarette filter. Only 51 (60%) of the respondents identified plastic (or plastic and paper) correctly. Between 94 and 98% of all respondents correctly identified that TPW is harmful to human health, natural environments, drinking water supplies, animals, and aquatic organisms.

### 3.3. Awareness and Perceptions

Regarding previously hearing the term “TPW”, 82% reported yes, but only 63% affirmed that, “I am well informed about TPW”. Further, the respondents were asked about their familiarity with the environmental principles of EPR and PS [20]. Overall, 58% of the respondents were unfamiliar with these principles, while 3% only reported familiarity with PS, 22% only reported familiarity with EPR, and 17% reported familiarity with both (2% did not respond).

Respondents were asked regardinf perceptions of prevention, reduction, and mitigation (PRM) strategies for TPW and its environmental impacts. All of the respondents concurred that adding a litter fee to fund TPW programs will aid in reducing tobacco use and the environmental impacts of TPW (Table 2). All but one agreed that PRM of TPW could be an important part of international tobacco control programs, and all but three agreed that banning smoking in outdoor venues could reduce TPW. Most did not concur that waste receptacles and pocket ashtrays were the most important part of PRM of TPW. Only 16% reported effective prevention or clean-up efforts in their countries.

The respondents were asked to rank the order in which groups, agencies, or organizations should be responsible for PRM of TPW (Table 3). Subsequently, we developed a linearly-weighted importance metric (decision matrix based on rankings). This metric is calculated for each policy option (*p_i_*), as shown in Equation (1), and it linearly weights the frequency and ranking importance.

(1) $$\overset{k}{\underset{i=1}{\sum }}\left(k+1-i\right)\times Rank_{i}/\overset{k}{\underset{i=1}{\sum }}k$$

In the case of this question, there are *k* = 9 rankings, so Rank 1 for policy option i is multiplied by 9, Rank 2 by 8, Rank 3 by 7, etc. Subsequently, these weighted rankings are divided by the sum of the ranks. In this way, a weighted importance value is assigned to each possible organization. The respondents believed that the national government (10.4 weighted ranking), tobacco industry (9.2 weighted ranking), and state governments (8.4 weighted ranking) should be largely responsible (Table 3).

All but one respondent agreed that EPR and PS should apply to PRM of TPW. Most of the respondents indicated that their organization did not engage with activities for PRM of TPW. Only 34% of the respondents had ever participated in TPW cleanups. Those who worked more than 10 years in tobacco control were more often involved in these efforts (43%) (Table 4).

The fact that many respondents (29%) incorrectly reported that filters did not make cigarettes easier to smoke was also of interest (question 5 part 4). In investigating this phenomenon, there were several interesting relationships that emerged. The relationship between this response and that of question 5 part 1, “I am well-informed about TPW” was evaluated by Fisher’s Exact Test (FET), a multivariate equivalent of the hypergeometric particularly appropriate when the cell counts are small and was statistically significant (*p* = 0.041). Surprisingly, nine of the 10 individuals who strongly disagreed (as well as two of the five who disagreed) with the statement that filters make cigarettes easier to smoke also strongly agreed that they were “well-informed about TPW.” Four out of eight of the individuals who strongly disagreed with question 5 part 4 also indicated that second-hand smoke was TPW in question 3 part 6. The Fisher’s Exact Test between these two questions was also statistically significant (*p* = 0.046). The respondents expressing familiarity with TPW also expressed familiarity with EPR and PS (FET, *p* < 0.03); however, no WHO regional effects were found (FET, *p* = 0.17). No regional effects were found for the 16% reporting effective prevention and clean-up of TPW in their countries either (FET, *p* = 0.13).

## 4. Discussion

This study suggests that, although progress has been made, there are still some significant knowledge gaps and opportunities for action on tobacco use and its environmental impacts, particularly regarding TPW. When comparing the results of the 2014 study to these results, knowledge of TPW by experts in the field is relatively strong and it has increased overall. There was an increase from 62% to 82% having ever heard of the term TPW and an increase from 29% to 63% stating that they felt they were “well informed about TPW” [8]. There was also improvement in knowedge regarding TPW being the most common items that are picked up during clean-up events, 64% in 2014 and 82% answering correctly in 2019. In this study, the majority of respondents answered correctly about the near lack of biodegradability of filters (88%), from the previous correct responses of only 73%. This increase in knowledge is an indicator that there is more concern and more interest in understanding the role of TPW in tobacco control and that educational campaigns are working [2,8].

The current study did however show two particular areas of weakness in knowledge that are of concern. First is the gap in knowledge of filter composition, with only 60% correctly answering and secondly, that 29% of respondents did not know that filters make smoking easier. Another concern was regarding the awareness of EPR and PS, which was poor among all of the respondents (57% were not at all familiar), similar to the results in 2014. All who reported that they were aware of both EPS and PS also agreed that they were very familiar with TPW. The lack of awareness regarding exactly what filters are made of and how they are problematic from a health perspective is an area that needs to be addressed among tobacco control experts [29]. Additionlly, more research and information on EPR and PS and its applicability to TPW is needed to help understand what industry accountability may be needed in preventing TPW from entering into the environment [7,9,20,21,22].

Only 16% reported that there were effective clean-up and prevention measures for TPW in their countries, and this did not vary by WHO Region. The respondents mostly favored adding a litter fee to fund TPW programs and largely agreed that the PRM of TPW could be an important part of international tobacco control programs. Most agreed that banning smoking in outdoor venues could reduce TPW. Most did not concur that waste receptacles and pocket ashtrays were the most important part of PRM of TPW. Only 34% of respondents had actually participated in cleanup efforts that are associated with cigarette butts. When ranking the responsibility for TPW, the government and tobacco industry were foremost, which was a change from the previous study that ranked the tobacco industry and smokers as the top responsible parties [8].

This research has several limitations. The sample size is small and it may have selection bias. The knowledge, perceptions, and awareness of the respondents may not reflect the entire FCA community. Inference from the responses should be appropriately caveated. The survey itself was short enough to encourage participation, which also limits what might be asked in more detail.

## 5. Conclusions

The opinions of the FCA members likely carry more weight with government agencies and decision makers, and may therefore have a larger influence on TPW control efforts. Understanding the knowledge, attitudes, and perceptions of these individuals is therefore important, as they help to shape important policies that can have global influence on our environment and society. Further, addressing TPW as a potential tobacco control intervention channel joins the tobacco control community with a potentially important set of allies in the environmental advocacy movement. TPW is the single most picked up item on beaches and urban cleanups globally, and it is hence a target for EPR and PS strategies. Articles 17 and 18 of the FCTC provide a vehicle through which the Conference of the Parties (COP) countries may directly address TPW as an environmental issue with national program obligations. This concept was at least considered at the FCTC COP 8 meeting in Geneva in 2018 [30], but it was referred for further study.

What is needed now is recognition that the cellulose acetate cigarette filter, which is attached to nearly all manufactured cigarettes sold globally, is simply a marketing tool with no health benefit, and which is the main component of the TPW problem that is now recognized by the tobacco control community as an environmental blight. It is highly likely that banning the sale of cigarettes with non-biodegradable or biodegradable filters will positively impact cessation, discourage the uptake of smoking by youth, and help to assign extended responsibility for TPW to the tobacco industry. It is also highly likely that reducing TPW will address existing environmental inequalities by reducing the environmental burden of TPW that differentially impacts communities where smoking is more common.

## Figures and Tables

**Table 1:** Knowledge about Tobacco Product Waste, Members of Framework Convention Alliance, 2019 (*N* = 65).

Question	*n* Correct (%)
1. Are cigarette butts TPW?	65 yes (100%)
2. Is tobacco product packaging TPW?	57 yes (88%)
3. Are plastic bags TPW?	48 no (74%)
4. Is electronic waste from e-cigarettes TPW?	30 yes (60%)
5. Are ashtrays TPW?	39 yes (60%)
6. Is 2d-hand smoke TPW?	48 no (74%)

TPW = tobacco product waste.

**Table 2:** Perception of prevention, reduction, and mitigation (PRM) strategies, members of Framework Convention Alliance, 2019 (*N* = 65).

Statement Regarding PRM	Strongly Disagree	Disagree	Agree	Strongly Agree	Don’t Know/No Response
1. PRM of TPW can be an important component of international tobacco control programs	0% (0)	1.5% (1)	12.3% (8)	83.1% (54)	3.1% (2)
2. Your organization includes TPW PRM as part of its tobacco control work	3.1% (2)	32.3% (21)	27.7% (18)	21.5% (14)	15.4% (10)
3. Addressing cigarette butts and other TPW can aid in reducing tobacco use	1.5% (1)	6.2% (4)	40.0% (26)	43.1% (28)	9.2% (6)
4. Banning the sale of filtered cigarettes can reduce the environmental impact of TPW	3.1% (2)	9.2% (6)	27.7% (18)	44.6% (29)	15.4% (10)
5. Adding a litter fee to fund TPW programs will aid in reducing tobacco use and the environmental impacts of TPW	0% (0)	0% (0)	30.8% (20)	60.0% (39)	9.2% (6)
6. Banning smoking in outdoor venues can reduce TPW	3.1% (2)	1.5% (1)	36.9% (24)	49.2% (32)	9.2% (6)
7. Waste receptacles and pocket ashtrays are the most important intervention to PRM TPW	21.5% (14)	23.1% (15)	29.2% (19)	10.7% (7)	15.4% (10)
8. There are effective TPW clean-up or prevention efforts in my country	43.1% (28)	27.7% (18)	12.3% (8)	1.5% (1)	15.4% (10)

**Table 3:** Rankings of organizational responsibility for tobacco product waste (TPW) (and ranking metric) by members of Framework Convention Alliance, 2019 (*N* = 65).

Responsible Party	Weighted Ranking in Descending Order
National Government	10.4
Tobacco Industry	9.2
State/Provincial Government	8.4
Local Government	8.1
Smokers	7.7
Environmental Groups	6
Communities	5.5
Tobacco Control Coalitions	5.3
Other	0.9

**Table 4:** TPW stewardship attitudes and practices by members of Framework Convention Alliance, 2019 (*N* = 65).

Statement	Strongly Disagree	Disagree	Agree	Strongly Agree	Don’t Know/No Response
1. EPR and PS should apply to PRM of TPW	0% (0)	1.5% (1)	16.9% (11)	60.0% (39)	21.5% (14)
2. The Framework convention on Tobacco Control includes opportunities for PRM of TPW and its impact	0% (0)	3.1% (2)	35.4% (23)	41.5% (27)	20.0% (13)
3. Our organization includes efforts to PRM of TPW as part of its tobacco control work	15.4% (10)	32.3% (21)	27.7% (18)	9.2% (6)	15.4% (10)
4. Our organization would be interested in learning more about TPW PRM as part of our tobacco control efforts	0% (0)	3.1% (2)	44.6% (29)	36.9% (24)	15.4% (10)

EPR = Extended Producer Responsibility.
